# Blended Acceptance and Commitment Therapy Versus Face-to-face Cognitive Behavioral Therapy for Older Adults With Anxiety Symptoms in Primary Care: Pragmatic Single-blind Cluster Randomized Trial

**DOI:** 10.2196/24366

**Published:** 2021-03-26

**Authors:** Maartje Witlox, Nadia Garnefski, Vivian Kraaij, Margot W M de Waal, Filip Smit, Ernst Bohlmeijer, Philip Spinhoven

**Affiliations:** 1 Faculty of Social and Behavioural Sciences Section of Clinical Psychology, Institute of Psychology Leiden University Leiden Netherlands; 2 Department Public Health & Primary Care Leiden University Medical Center Leiden Netherlands; 3 Department of Mental Health & Prevention Netherlands Institute of Mental Health and Addiction Utrecht Netherlands; 4 Department of Clinical, Neuro and Developmental Psychology Vrije Universiteit Amsterdam Netherlands; 5 Department of Epidemiology & Biostatistics Amsterdam University Medical Centers VU University Medical Center Amsterdam Netherlands; 6 Department of Psychology, Health and Technology University of Twente Enschede Netherlands

**Keywords:** acceptance and commitment therapy, anxiety, older adults, internet interventions, cognitive behavioral therapy

## Abstract

**Background:**

Anxiety symptoms in older adults are prevalent and disabling but often go untreated. Most trials on psychological interventions for anxiety in later life have examined the effectiveness of face-to-face cognitive behavioral therapy (CBT). To bridge the current treatment gap, other treatment approaches and delivery formats should also be evaluated.

**Objective:**

This study is the first to examine the effectiveness of a brief blended acceptance and commitment therapy (ACT) intervention for older adults with anxiety symptoms, compared with a face-to-face CBT intervention.

**Methods:**

Adults aged between 55-75 years (n=314) with mild to moderately severe anxiety symptoms were recruited from general practices and cluster randomized to either blended ACT or face-to-face CBT. Assessments were performed at baseline (T0), posttreatment (T1), and at 6- and 12-month follow-ups (T2 and T3, respectively). The primary outcome was anxiety symptom severity (Generalized Anxiety Disorder-7). Secondary outcomes were positive mental health, depression symptom severity, functional impairment, presence of Diagnostic and Statistical Manual of Mental Disorders V anxiety disorders, and treatment satisfaction.

**Results:**

Conditions did not differ significantly regarding changes in anxiety symptom severity during the study period (T0-T1: B=.18, *P*=.73; T1-T2: B=−.63, *P*=.26; T1-T3: B=−.33, *P*=.59). Large reductions in anxiety symptom severity (Cohen *d*≥0.96) were found in both conditions post treatment, and these were maintained at the 12-month follow-up. The rates of clinically significant changes in anxiety symptoms were also not different for the blended ACT group and CBT group (χ^2^_1_=0.2, *P*=.68). Regarding secondary outcomes, long-term effects on positive mental health were significantly stronger in the blended ACT group (B=.27, *P*=.03, Cohen *d*=0.29), and treatment satisfaction was significantly higher for blended ACT than CBT (B=3.19, *P*<.001, Cohen *d*=0.78). No other differences between the conditions were observed in the secondary outcomes.

**Conclusions:**

The results show that blended ACT is a valuable treatment alternative to CBT for anxiety in later life.

**Trial Registration:**

Netherlands Trial Register TRIAL NL6131 (NTR6270); https://www.trialregister.nl/trial/6131

## Introduction

### Background

Anxiety is among the most common mental health problems in older adults, with prevalence estimates for anxiety disorders ranging up to 15% [1-3]. When also considering the presence of anxiety symptoms that do not meet the diagnostic criteria for a disorder (so-called subclinical or subthreshold anxiety), estimates range between 15% and 52% [1,3]. Both anxiety disorders and subclinical anxiety in older adults are associated with limited physical and social activities, impairments in self-care, decreased well-being, comorbid depressive symptomatology, somatic problems, and increased use of benzodiazepines [4-6]. Despite the repeatedly demonstrated negative impact of anxiety in later life, only a small proportion of anxious older adults receive adequate psychological help [7-9]. This treatment gap is worrying as untreated anxiety symptoms in older adults tend to be chronic and aggravated over time [10].

The current scientific literature on psychological interventions for anxiety in later life is limited with regard to both well-evaluated treatment approaches and the precise types of anxiety they target. The large majority of trials in anxious older adults have investigated face-to-face cognitive behavioral therapy (CBT) for Generalized Anxiety Disorder (GAD). In the most recent meta-analysis on CBT for anxiety disorders in older adults that concluded CBT to be an effective treatment, 7 of the 12 included studies focused on GAD [11]. In recent years, researchers’ focus has shifted a little to web-based and blended CBT interventions as treatment for anxiety in later life. To date, studies in older adults with heterogeneous anxiety symptomatology have found web-based CBT modules combined with guidance from a clinician to be effective in reducing symptom severity [12-15]. These results are promising, as scalable (partly) web-based interventions might be invaluable in bridging the current treatment gap in a cost-effective way.

As CBT is the only treatment that has been systematically studied and most studies thus far confirm its effectiveness, many clinical guidelines refer to it as the preferred treatment for older adults with anxiety [16-18]. However, to move the field forward and improve treatment of anxiety in later life, alternative treatment options should also be evaluated, because in most studies with active control conditions, effect sizes favoring CBT were small [11], and some evidence suggests that older adults benefit less from CBT for anxiety than younger adults [11,19,20]. It has been hypothesized that the cognitive aspects of challenging negative thoughts could be especially problematic for older adults [20]. Unfortunately, no high-quality studies on other treatment approaches have yet been published.

Acceptance and commitment therapy (ACT), a promising alternative to CBT, has been found to be effective in reducing anxiety symptoms in general adult samples, both in face-to-face and (partly) web-based formats [21,22]. Contrary to CBT, which focuses on re-evaluating cognitions and changing safety behavior and avoidance to achieve decreased levels of anxiety, ACT promotes acceptance-based emotion regulation and valued engagement in life [23]. ACT ultimately aims to increase psychological flexibility: the ability to fully and openly experience the present moment, including the negative aspects, and to behave in accordance with personal values [23]. It has been recognized as a treatment that explicitly aligns with the understanding of mental health as not only the absence of disease and illness but also the presence of the so-called positive mental health [24-26].

ACT might be especially suitable for older adults because its focus on stimulating acceptance and value-based action is consistent with age-related changes in emotion regulation and behavior. Reorientation on personal values and associated behavior change [27,28], present moment awareness, and willingness to experience and accept negative emotions have all been found to increase with age [29,30]. As some studies suggest that treatment is more effective when it draws upon a patient’s strengths rather than remediating their shortcomings [31,32], ACT holds promise as a particularly suitable treatment approach for older adults. Another argument for ACT as a treatment option for anxiety in later life is its transdiagnostic focus on increasing psychological flexibility. Low levels of psychological flexibility have been related to both anxiety and depression symptoms [33], which often co-occur in older adults. Although ACT seems to be a promising treatment option for older adults with anxiety, so far only one pilot study that examined face-to-face ACT for late-life GAD has been published. None of the participants dropped out and worry and depression scores improved [28], leading the authors to conclude that ACT warrants a large-scale evaluation in anxious older adults.

### Objectives

This trial aims to advance evidence-based treatment of anxiety in later life by evaluating the short- and long-term effectiveness of an ACT intervention in a large sample of older adults with anxiety symptoms. Specifically, we will evaluate a blended ACT intervention, because scalable internet-based interventions could be crucial in bridging the treatment gap in anxious older adults and should therefore be thoroughly evaluated. Furthermore, the low-threshold nature and easy accessibility of internet-based interventions might be especially appealing to older adults, who are known to experience barriers in seeking and receiving regular psychological treatment [9]. The blended ACT intervention will be compared with a face-to-face CBT intervention, which can be considered treatment as usual in the study setting [34-36]. As the ACT approach aligns with age-related changes in emotion regulation and behavior, we expect the ACT intervention to be more effective than CBT. In addition to the effect on the primary outcome anxiety symptom severity, the effects of interventions on positive mental health, depressive symptoms, functional impairment, presence of anxiety disorders, and treatment satisfaction will be evaluated. As this study is the first large-scale trial into an ACT intervention for anxiety in later life, the results will offer valuable new insights into how the large and currently underserved group of older adults with anxiety symptoms can be treated.

## Methods

### Design

The study was registered in the Netherlands Trial Register (NL6131; NTR6270) and approved by the Medical Ethics Committee of Leiden University Medical Center (P16.248). A detailed description of the study protocol has been published [37].

The study was designed as a pragmatic, single-blind cluster, randomized controlled trial with measurements at baseline (T0) and follow-ups at 3, 6, and 12 months (T1, T2, T3, respectively) postbaseline. Randomization took place at the level of mental health counselors working in general practices, creating clusters of participants who received treatment from the same counselor. Power analysis showed that to detect a between-group difference on the Generalized Anxiety Disorder-7 (GAD-7) at posttreatment with a medium effect size (Cohen *d*=0.45), a 2-tailed α of .05, and a power of 0.80 [38], posttreatment data of 180 participants were required. Anticipating a dropout rate of 25%, 240 participants (36 counselors) were included at baseline.

The block-randomization table (blocks of 4) was created by an independent researcher using the *R* software [38] and was concealed from the main researcher. If 4 mental health counselors had registered for participation, the main researcher received their allocation from an independent researcher. After a mental health counselor was informed about their randomization status and had received training in the treatment they were allocated to provide to study participants, recruitment of participants from the general practice that employed the counselor started.

Research assistants (Master’s students or graduates in clinical psychology) who conducted telephonic diagnostic interviews as part of the assessments were blinded to the participants’ treatment assignments. The main researcher, mental health counselors, and participants were not blinded to treatment allocation. Study participants were not informed whether the intervention they received was the experimental or the active control condition. To prevent selection bias, potential participants were not informed about the randomization status of the mental health counselor in their general practice (ie, the intervention they would receive if they participated in the study) until they had given their informed consent and completed the baseline assessment.

### Study Setting: General Practices

The treatment was provided by mental health counselors working in general practices in the Netherlands. Since 2008, general practices in the Netherlands have employed mental health counselors in response to the increasing demand for psychological treatment and the limited capacity of mental health care institutions [34]. The counselors offer brief psychological interventions to patients with mild to moderately severe symptomatology in the easily accessible environment of general practices.

General practices were recruited by sending information and invitation letters to practices in the networks of Leiden University and Leiden University Medical Centre. Furthermore, study information was distributed through messages in relevant newsletters and online forums. When a general practice agreed upon study participation, employees of the practice were asked to distribute the information among their professional networks. A total of 38 general practices were recruited. These practices were located in villages (n=10), towns (n=11), and cities (n=17) throughout the Netherlands, in 9 out of 12 provinces. The practices employed a total of 40 mental health counselors, who were randomized to provide study participants with either blended ACT (n=20) or face-to-face CBT (n=20). In total, 36 practices employed one mental health counselor and 2 practices employed 2 counselors each. Regarding the counselors’ educational background, most were psychologists (n=13), social psychiatric nurses (n=14), or social workers (n=5). Two counselors were trained as system therapists, and the other 6 had different educational backgrounds. The number of years of experience in providing individual psychological treatment ranged from 3 to 42, with a median of 16 years.

### Participants

Individuals aged between 55-75 years with mild to moderately severe anxiety symptoms (GAD-7 between 5 and 15 [39]) were eligible for participation. Mastery of the Dutch language, internet access, and motivation to spend 2.5 h per week on the intervention were also required. Exclusion criteria were severe cognitive impairment or unstable severe medical conditions (according to the medical record at the general practice); very mild or severe anxiety symptoms (GAD-7 score <5 or >15 [39]); severe depressive symptomatology (Patient Health Questionnaire-9 [PHQ-9] score≥20 [40]), psychological or psychopharmacological treatment within the last 3 months, with the exception of stable benzodiazepine or selective serotonin reuptake inhibitor use; severe functional impairment (score≥8 on 2 or 3 Sheehan Disability Scale (SDS) domains [41]), high suicide risk (Mini-International Neuropsychiatric Interview Plus [MINI-Plus]) [42]; substance use disorder (MINI-Plus); lifetime diagnosis of bipolar disorder or schizophrenia (medical record or MINI-Plus).

### Procedure

Patients (aged between 55 and 75 years) from participating general practices were sent a letter containing information about anxiety symptoms, the aim and design of the study, and an invitation to participate. A data manager from the Leiden University Medical Center assisted general practitioners (GPs) in preparing and sending the letters in accordance with Dutch privacy legislation. Patients whose medical records mentioned a lifetime diagnosis of bipolar disorder or schizophrenia, severe unstable medical conditions, or severe cognitive impairment did not receive an invitation letter. GPs could also exclude patients from the mailing list for other reasons (eg, social circumstances or language barriers) and had to give written approval of the final mailing list.

The information or invitation letters refer people to the study website for detailed information about the trial and to register for participation. After registration, they were screened using web-based questionnaires (assessing anxiety severity [GAD-7], depression severity [PHQ-9], mastery of Dutch, and motivation for treatment) and by a telephone interview (assessing medication use, functional impairment [SDS], and presence of psychiatric disorders [MINI-Plus]). If excluded for the presence of severe symptomatology, people were referred to their GP to discuss other treatment options. Web-based informed consent was obtained from all eligible participants before they completed the web-based baseline questionnaire. After this, the main researcher informed the included participants about the intervention they would receive and updated the general practice about the inclusion.

Participants completed 4 assessments (T0, T1, T2, and T3). Assessments mainly consisted of web-based self-report questionnaires. Assessments at T0, T1, and T3 were complemented by telephone interviews conducted by trained research assistants.

### Treatments

#### Blended ACT

Participants in the blended ACT condition were given access to the web-based ACT-module *Living to the Full* and attended 4 face-to-face sessions with their mental health counselor at the general practice. The *Living to the Full* module consisted of 9 lessons to be completed in 9 to 12 weeks. This module (an adaptation of the similarly titled self-help book [43,44]) was proven effective in reducing distress and depression in earlier studies [45,46]. The web-based module could be accessed using computers and mobile devices. To complete the lessons in time, the participants were required to spend 15 minutes to 30 minutes on the module each day. The module consisted of 3 phases, each comprising 3 lessons. In the first phase, participants explored the negative consequences of their attempts to control or reduce their unwanted feelings or thoughts and were introduced to the idea of shifting their attitude toward their internal experiences from controlling to accepting. The next 3 lessons provided them with tools to be more accepting of their (unwanted) internal experiences: exercises focused on noticing thoughts and feelings without judgment and conceptualizing the self as the consciousness that notices internal experiences, instead of the content of these experiences. The last phase of the module focused on identifying core values and taking the first step toward living in accordance with these.

The authors of *Living to the Full* developed a treatment protocol for the 4 face-to-face sessions with the mental health counselor at the general practice. In the first session, the participants’ complaints were inventoried and a web-based program was introduced. After this session, the participants were emailed their log-in credentials and could access the web-based module. The subsequent 3 lessons each connected to 1 of the 3 phases in the module and served to repeat key exercises, increase motivation, evaluate progress, and discuss potential problems. Mental health counselors could monitor the progress of their clients in the web-based module: they could see their answers to the exercises and the amount of time they spent on the module but could not provide web-based feedback.

#### Treatment-As-Usual: Face-to-face CBT

Participants in the treatment-as-usual group received a protocolized CBT intervention, consisting of 4 face-to-face sessions over a period of 9 to 12 weeks. In addition, participants were given homework exercises that required 15 to 30 min per day (ie, a similar time investment as the blended ACT intervention). The treatment protocol was developed by NG, MW, VK, and PS. It consisted of a manual with 12 different worksheets containing psychoeducation and CBT exercises. The main worksheets focused on thinking errors and avoidance behaviors. Other worksheets addressed specific forms of anxiety (eg, worrying, panic, social anxiety) or common consequences of anxiety (eg, sleep disturbances, muscle tension). On the basis of the intake and goal formulation during the first session, counselors and participants agreed upon which worksheets to use. In Session 2 and 3, the mental health counselor and participant discussed and repeated homework exercises, evaluated progress, and discussed potential problems, and the counselor aimed to increase the participants’ motivation to continue with the intervention. The last session was dedicated to formulating a relapse prevention plan.

Mental health counselors received a 6-h long in-person training on working with the treatment protocol for their allocated treatment.

### Measures

Table 1 presents an overview of the instruments used per measurement moment. Anxiety symptom severity was assessed using the GAD-7 (total scores 0-21), with higher scores indicating higher symptom severity [39]. Positive mental health was measured using the Mental Health Continuum-Short Form (MHC-SF; total scores: (range 0-5) were obtained by averaging the sum scores of the 14 6-point items, with higher scores indicating higher levels of positive mental health [47]). Depressive symptoms were assessed using the PHQ-9 (total score 0-27; higher scores reflect higher symptom severity [40]). The SDS [41] assessed functional impairment in the domains of work, social life, and family life (scores in each domain range 0-10, higher scores reflecting more impairment). The presence of current GAD, panic disorder, agoraphobia, specific phobia, social phobia, obsessive-compulsive disorder, posttraumatic stress disorder, and illness anxiety disorder according to DSM-V criteria was assessed using the MINI-Plus [42]. Treatment satisfaction was assessed using the Client Satisfaction Questionnaire-8 (total scores 0-32; higher scores indicate higher satisfaction [48]). To assess treatment integrity, mental health counselors, after every session, indicated how closely they had followed the treatment protocol on a checklist with all the elements the protocol prescribed for the sessions. Secondary outcomes not reported in this article were mindfulness, experiential avoidance, cognitive emotion regulation, medical costs, and quality of life. These outcomes will be used in subsequent studies on moderator, mediator, and cost-effectiveness analyses.

**Table 1:** Instruments per measurement moment.

Instrument	Screening	T0^a^	T1^b^ (3 month)	T2 (6 month)	T3 (12 month)
Anxiety symptom severity (GAD-7^c^)	✓^d^	✓	✓	✓	✓
Positive mental health (MHC-SF^e^)	—^f^	✓	✓	✓	✓
Depression symptom severity (PHQ-9^g^)	✓	✓	✓	✓	✓
Presence of psychiatric disorders^h^ (MINI-Plus^i^)	✓	✓	✓	—	✓
Functional impairment^h^ (SDS^j^)	✓	✓	✓	—	✓
Treatment satisfaction (CSQ-8^k^)	—	—	✓	—	—

^a^T0: baseline.

^b^T1: posttreatment.

^c^GAD-7: Generalized Anxiety Disorder-7.

^d^Indicates that the instrument was used at the specified measurement moment.

^e^MHC-SF: Mental Health Continuum-Short Form.

^f^Indicates that the instrument was not used at the specified measurement moment.

^g^PHQ-9: Patient Health Questionnaire-9.

^h^Assessed during telephone interviews by trained research assistants. Scores on these measures obtained during screening are analyzed as part of baseline.

^i^MINI-Plus: Mini-International Neuropsychiatric Interview-Plus.

^j^SDS: Sheehan Disability Scale.

^k^CSQ-8: Client Satisfaction Questionnaire-8.

### Statistical Analyses

Statistical analyses were performed using the *R* software [38]. The differences between conditions over time on continuous outcomes were examined using linear mixed models. The time variable was recoded into 3 contrasts: T0-T1 (baseline to posttreatment), T1-T2 (posttreatment to 6-month follow-up), and T1-T3 (posttreatment to 12-month follow-up). Functional impairment was not assessed at T2; therefore, these analyses included 2 contrasts (T0-T1 and T1-T3). The condition variable was effect-coded (CBT=−0.5, ACT=0.5) to ensure that the coefficients for the time variables reflected true main effects. Time, condition, and their interaction were included as fixed effects. Random intercepts were included at the participant level and mental health counselor level. Random slopes for time were included for mental health counselors but not for participants, as this would result in more parameters than observations. Treatment satisfaction was only assessed at T1, so this model included no time effects and only a random intercept at the counselor level. For this model, the condition was dummy coded (CBT=0, ACT=1).

Mixed effects logistic regression was used to examine if proportions of participants that changed from *anxiety disorder* to *no anxiety disorder*—and vice versa—differed between groups. A total of 4 separate models were created to examine the differences between the conditions at T1 and T3 for participants without an anxiety disorder. All mixed models were fitted to the data with the maximum likelihood. This method does not replace or impute missing values but uses all observed data to estimate the value of a population parameter by determining the value that maximizes the likelihood function [49].

Cohen *d* was used as the effect size for continuous outcomes and was calculated using mixed model estimated means and observed SD [50]. Cohen *d* values were interpreted as very small (<0.20), small (0.20-0.50), medium (0.50-0.80), or large (>0.80) [51]. Odds ratios were used as effect sizes for between-group differences on the binary outcome and were classified as small (1.49-3.45), medium (3.45-9), and large (>9) [52].

For participants with a GAD-7 posttreatment score, a reliable change index (RCI) was calculated by dividing the difference between baseline and posttreatment scores by the standard error of difference (SED) [52]. The test-retest reliability of the GAD-7 (0.83) was used to calculate the SED [39]. RCI values lower than −1.96 indicate reliable symptom improvement, and values over 1.96 denote deterioration [53]. Recovery was operationalized as a posttreatment score below the cut-off for moderately severe anxiety symptoms (GAD-7<10 [40]) for participants who scored above this cut-off at baseline. Participants with both reliable improvement and recovery met the criteria for clinically significant changes [53]. The proportions of participants with reliable improvement, deterioration, and clinically significant change in both groups were compared using the *χ*^2^ test.

In addition to intention-to-treat (ITT) analyses, per-protocol (PP) analyses were also conducted. For both groups, PP treatment was defined as attending 3 or 4 (75% or more of the allocated treatment) of the face-to-face sessions.

## Results

### Participants

**Figure 1:**
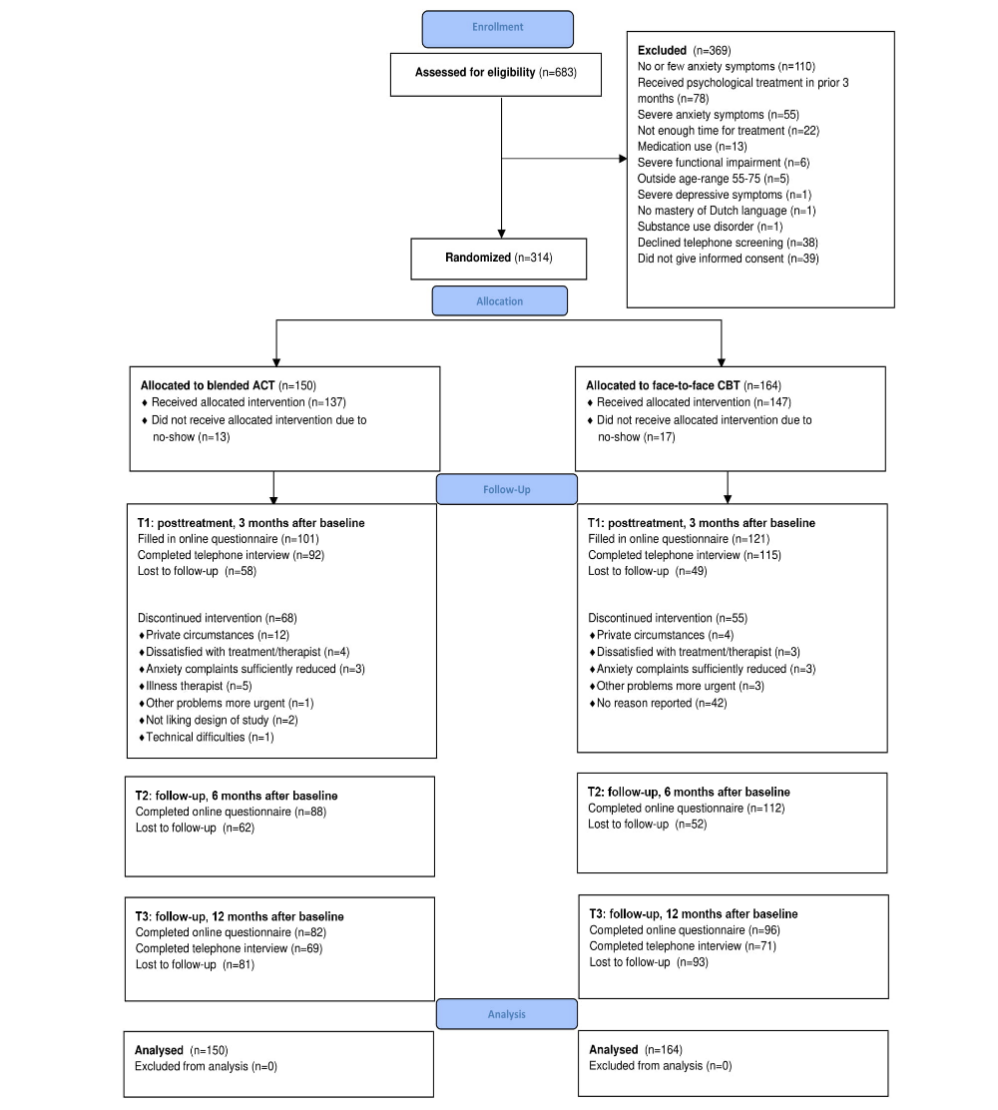
Flowchart of study participants. ACT: acceptance and commitment therapy; CBT: cognitive behavioral therapy.

Figure 1 presents the flowchart of the participants. From November 2017 to March 2019, 35,820 invitation letters were sent. A total of 683 people were screened, of whom 314 were included: 150 in the blended ACT group and 164 in the CBT group. Table 2 shows the demographic and clinical characteristics of the participants. A total of 13 participants in the ACT group and 17 in the CBT group did not start the treatment, as they did not show up for the first appointment and later indicated that they wanted to stop their participation or were not reachable by phone and email to discuss further participation. At T1, 70.7% (222/314) of the participants completed the web-based questionnaire (ACT 101/150, 67.3%, CBT 121/164, 73.8%); at T2, 63.7% (200/314; ACT 88/150, 58.6%, CBT 112/164, 68.3%), and at T3, 56.7% (178/314; ACT 82/150, 55%, CBT 96/164, 59%). Telephone interviews at T1 and T3 were completed by 66% (208/314; ACT 92/150, 61.3%, CBT 115/164, 70.1%) and 44.6% (140/314; ACT 69/150, 46.0%; CBT 71/164, 43.3%), respectively.

**Table 2:** Baseline characteristics of study sample.

Characteristics		Blended ACT^a^ (n=150)	CBT^b^ (n=164)	Total sample (n=314)
**Age (years)**				
	Mean (SD)	62.75 (5.69)	63.33 (5.71)	63.06 (5.70)
	Range	55-75	55-75	55-75
**Sex, n (%)**				
	Female	100 (66.7)	92 (56.1)	192 (61.2)
	Male	50 (33.3)	72 (43.9)	122 (38.9)
**Nationality, n (%)**				
	Dutch	149 (99.3)	159 (96.9)	308 (98.0)
	Dutch and other	0 (0.0)	5 (3.0)	5 (1.6)
	Other	1 (0.7)	0 (0.0)	1 (0.4)
**Education, n (%)**				
	Low	22 (14.7)	15 (9.2)	37 (11.7)
	Middle	70 (44.7)	74 (45.1)	144 (45.8)
	High	56 (37.3)	74 (45.1)	130 (41.4)
	Unknown	2 (0.6)	1 (0.6)	3 (0.9)
**Relational status, n (%)**				
	Married or in a romantic relationship	120 (80.0)	129 (78.6)	249 (79.3)
	Not married or in a romantic relationship	30 (20.0)	35 (21.3)	65 (20.7)
**Work status, n (%)**				
	Paid employment	77 (51.3)	76 (46.3)	153 (48.7)
	Voluntary work	49 (32.6)	56 (34.1)	105 (33.4)
	No work	53 (35.3)	59 (35.9)	112 (35.6)
**Living situation, n (%)**				
	Alone	36 (24.0)	39 (23.8)	75 (23.9)
	With partner	97 (64.6)	103 (62.8)	200 (63.7)
	With children	11 (7.3)	13 (7.9)	24 (7.6)
	With partner and children	6 (4.0)	8 (4.8)	14 (4.5)
	Other	0 (0.0)	1 (0.6)	1 (0.3)
	Community-dwelling	150 (100.0)	164 (100.0)	314 (100.0)
**Somatic comorbidity, n (%)**				
	No somatic problems	29 (19.3)	32 (19.5)	61 (19.4)
	One or more somatic problems	121 (80.7)	132 (80.5)	253 (80.6)
**Psychomedication use, n (%)**				
	SSRI^c^	10 (6.7)	12 (7.3)	22 (7.0)
	Benzodiazepine	19 (12.7)	15 (9.2)	34 (10.8)
	No psychotropic medication	121 (80.7)	137 (83.5)	258 (82.2)
**Anxiety disorder, n (%)**				
	Panic disorder	10 (6.7)	7 (4.3)	17 (5.4)
	Agoraphobia	5 (3.3)	5 (3.0)	10 (3.2)
	Social phobia	5 (3.3)	8 (4.9)	13 (4.1)
	Specific phobia	10 (6.7)	8 (4.8)	18 (5.7)
	OCD^d^	1 (0.7)	2 (1.2)	3 (0.9)
	PTSD^e^	2 (1.3)	1 (0.6)	3 (0.9)
	Illness anxiety disorder	3 (2.0)	4 (2.4)	7 (2.2)
	GAD^f^	17 (11.3)	18 (10.9)	35 (11.1)
	Any anxiety disorder	42 (28.0)	39 (23.8)	81 (25.8)
	No anxiety disorder	108 (72.0)	125 (76.2)	233 (74.2)

^a^ACT: acceptance and commitment therapy.

^b^CBT: cognitive behavioral therapy.

^c^SSRI: selective serotonin reuptake inhibitor.

^d^OCD: obsessive-compulsive disorder.

^e^PTSD: posttraumatic stress disorder.

^f^GAD: generalized anxiety disorder.

### Treatment Adherence and Study Dropout

Of the 314 participants, a total of 191 (60.8%) attended all 4 face-to-face sessions and 35 (11.1%) attended 3 sessions. Significantly more participants attended 3 or 4 sessions (ie, received PP treatment) in the CBT group than in the ACT group (CBT: 126/164, 76.8%, ACT: 100/150, 66.7%, (*χ*^2^_1_=4.0, *P=*.045). A total of 41 participants reported their reason for dropping out of treatment (Figure 1).

The proportion of participants who completed the T1 measurement did not differ between the groups (*χ*²_1_=1.6, *P=*.21). Baseline characteristics did not differ significantly between participants who completed T1 and those who did not. Of the 222 participants who completed T1, 201 (90.5%) attended either 3 or 4 face-to-face sessions. There was no difference between the groups regarding the time participants at T1 reported to have spent on homework exercises or completing the web-based module (*F_1_*=1.239; *P=*.27).

### Treatment Integrity

Mental health counselors in the ACT and CBT groups completed the treatment integrity checklist for 71.1% (315/443) and 82% (424/517) of the sessions, respectively. The ACT group indicated adherence to all the prescribed elements for 80% (252/315) of the sessions. For the CBT group, this was 85.8% (364/424) of the sessions.

#### Primary Outcomes

**Figure 2:**
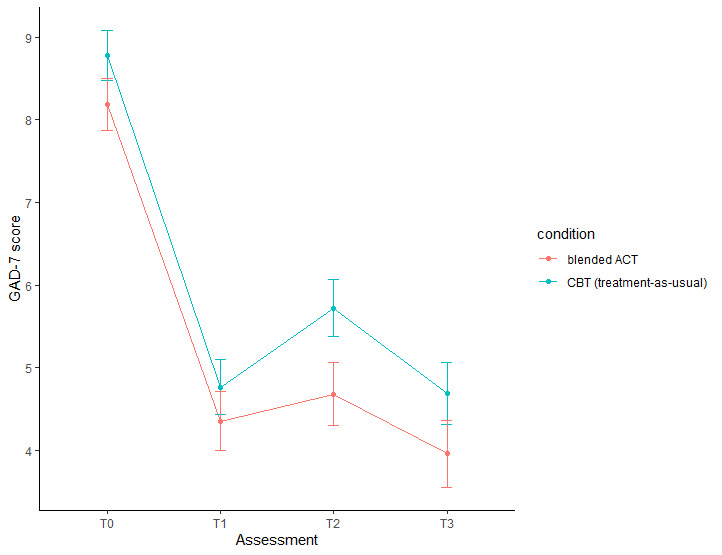
Mean Generalized Anxiety Disorder-7 (GAD-7) scores at all assessments for both conditions. ACT: acceptance and commitment therapy; CBT: cognitive behavioral therapy; GAD-7: Generalized Anxiety Disorder-7.

Tables 3 and 4 contain the results of the mixed models and the models’ estimated mean scores. Figure 2 presents the estimated mean GAD-7 scores for all measurement moments for the 2 groups. Regardless of the condition, GAD-7 scores significantly decreased from T0 to T1 (B=−3.92, *P<*.001), increased significantly between T1 and T2 (B=.64, *P=*.02), and did not change significantly from T1 to T3 (B=−.23, *P=*.45). The within-group effect sizes for both conditions were large for the decreases from T0 to T1 (ACT: Cohen *d*=0.96; CBT: Cohen *d*=1.09) and small to very small for T1-T2 (ACT: Cohen *d*=0.10; CBT: Cohen *d*=0.28) and T1-T3 (ACT: Cohen *d*=0.11; CBT: Cohen *d*=0.02) changes. All time-by-condition interactions were statistically insignificant, indicating that changes in anxiety symptom severity over time did not differ between the groups.

**Table 3:** Mixed model analyses comparing the differences between the blended acceptance and commitment therapy and cognitive behavioral therapy group over time and between-group effect sizesa.

Outcome		Unstandardized beta coefficient B		SE		*t* test (*df*)		*P* value		Cohen *d*	
**GAD-7^b^**											
	T0-T1		−3.92		0.26		−15.01 (57)		<.001		N/A^c^
	T1-T2		.64		0.28		2.29 (580)		.02		N/A
	T1-T3		−0.23		0.30		−0.78 (20)		.45		N/A
	T0-T1^d^ condition		.18		0.52		0.35 (57)		.73		0.02
	T1-T2^d^ condition		−0.63		0.56		−1.13 (580)		.26		0.15
	T1-T3^d^ condition		−0.33		0.60		−0.54 (20)		.59		0.08
**MHC-SF^e^**											
	T0-T1		.29		0.05		4.55 (34)		<.001		N/A
	T1-T2		.00		0.06		0.01 (173)		.99		N/A
	T1-T3		−0.06		0.06		−0.90 (71)		.37		N/A
	T0-T1^d^ condition		−0.12		0.13		−0.94 (34)		.36		0.06
	T1-T2^d^ condition		.03		0.12		0.24 (173)		.82		0.03
	T1-T3^d^ condition		.27		0.13		2.13 (71)		.04		0.29
**PHQ-9^f^**											
	T0-T1		−3.01		0.26		−11.59 (30)		<.001		N/A
	T1-T2		−0.65		0.27		−2.37 (66)		.02		N/A
	T1-T3		−0.69		0.33		−2.12 (42)		.04		N/A
	T0-T1^d^ condition		.31		0.52		0.59 (30)		.56		0.03
	T1-T2^d^ condition		−0.67		0.55		−1.21 (66)		.23		0.16
	T1-T3^d^ condition		−0.53		0.66		−0.80 (42)		.43		0.12
**SDS^g^ work**											
	T0-T1		−1.87		0.27		−6.96 (37)		<.001		N/A
	T1-T3		−.18		0.31		−0.58 (45)		.57		N/A
	T0-T1^d^ condition		.28		0.54		0.53 (37)		.60		0.10
	T1-T3^d^ condition		.64		0.62		1.03 (45)		.31		0.23
**SDS social life**											
	T0-T1		−1.78		0.26		−6.96 (37)		<.001		N/A
	T1-T3		−.15		0.27		−0.55 (27)		.59		N/A
	T0-T1^d^ condition		−.18		0.51		−0.35 (37)		.73		0.07
	T1-T3^d^ condition		0.08		0.55		0.15 (27)		.88		0.03
**SDS family and/or home**											
	T0-T1		−1.93		0.22		−8.78 (44)		<.001		N/A
	T1-T3		−.17		0.26		−0.66 (73)		.51		N/A
	T0-T1^d^ condition		.02		0.44		0.05 (44)		.96		0.00
	T1-T3^d^ condition		−.38		0.51		−0.74 (73)		.46		0.11
**CSQ-8^h^**											
	T1 Intercept		22.83		0.35		65.20 (34)		<.001		N/A
	T1 Condition		3.19		0.70		4.58 (37)		<.001		0.78
**MINI-Plus^i^ (for subgroup without anxiety disorder at baseline)^d^**											
	T1 Intercept		−3.47		0.96		−3.60^j^		<.001		N/A
	T1 Condition		1.28		0.78		1.64^j^		.10		3.59
	T3 Intercept		−2.38		0.47		−5.09^j^		<.001		N/A
	T3 condition		.05		0.70		0.07^j^		.941		1.05
**MINI-Plus (for subgroup with anxiety disorder at baseline)^d^**											
	T1 intercept		−1.34		0.46		−2.93^j^		.003		N/A
	T1 condition		.38		0.62		0.61^j^		.54		1.46
	T3 intercept		−1.39		0.79		−1.75^j^		.08		N/A
	T3 condition		−1.01		1.08		−0.94^j^		.35		2.75

^a^For the MINI-Plus and CSQ-8, the condition variable was dummy coded (CBT=0, ACT=1). B is the unstandardized coefficient. T0 stands for baseline; T1 for posttreatment; T2 and T3 for 6- and 12-month follow-up, respectively.

^b^GAD-7: Generalized Anxiety Disorder-7.

^c^N/A: not applicable.

^d^The presented b-coefficients of logistic mixed model regressions are log-its and effect sizes odds ratios.

^e^MHC-SF: Mental Health Continuum-Short Form.

^f^PHQ-9: Patient Health Questionnaire-9.

^g^SDS: Sheehan Disability Scale.

^h^CSQ-8: Client Satisfaction Questionnaire-8.

^i^MINI-Plus: Mini International Neuropsychiatric Interview-Plus.

^j^Reported values are z-values.

**Table 4:** Mixed model estimated means for the outcomes in both groups and within-group effect sizes.

Outcome		T0 baseline (95% CI)		T1 posttreatment (95% CI)		T2 6-month follow-up (95% CI)		T3 12-month follow-up (95% CI)		ES^a^ T0-T1		ES T1-T2		ES T1-T3	
**GAD-7^b^**															
	Blended ACT^c^		8.18 (7.49-8.88)		4.35 (3.59-5.12)		4.67 (3.86-5.49)		3.96 (3.09-4.83)		0.96		0.10		0.11
	CBT^d^		8.78 (8.12-9.44)		4.76 (4.06-5.47)		5.72 (4.99-6.45)		4.70 (3.89-5.50)		1.09		0.28		0.02
**MHC-SF^e^**															
	Blended ACT		2.73 (2.54-2.91)		2.96 (2.75-3.17)		2.98 (2.76-3.19)		3.04 (2.82-3.26)		0.24		0.02		0.09
	CBT		2.57 (2.40-2.74)		2.92 (2.73-3.12)		2.91 (2.72-3.10)		2.73 (2.52-2.94)		0.38		0.01		0.20
**PHQ-9^f^**															
	Blended ACT		6.99 (6.28-7.71)		4.14 (3.30-5.00)		3.16 (2.35-3.97)		3.19 (2.3-4.06)		0.70		0.26		0.27
	CBT		7.92 (7.24-8.60)		4.76 (3.97-5.55)		4.44 (3.71-5.18)		4.33 (3.52-5.14)		0.75		0.08		0.12
**SDS^g^ work**															
	Blended ACT		3.52 (2.94-4.11)		1.80 (1.16-2.44)		N/A^h^		1.94 (1.17-2.71)		0.67		N/A		0.06
	CBT		3.76 (3.17-4.35)		1.75 (1.17-2.34)		N/A		1.25 (0.45-2.05)		0.82		N/A		0.24
**SDS social life**															
	Blended ACT		4.02 (3.51-4.53)		2.16 (1.57-2.74)		N/A		2.05 (1.38-2.72)		0.75		N/A		0.04
	CBT		4.08 (3.59-4.56)		2.39 (1.86-2.91)		N/A		2.20 (1.53-2.86)		0.63		N/A		0.07
**SDS family and/or home**															
	Blended ACT		3.82 (3.30-4.33)		1.90 (1.34-2.45)		N/A		1.54 (0.84-2.23)		0.76		N/A		0.16
	CBT		3.79 (3.30-4.28)		1.85 (1.35-2.35)		N/A		1.87 (1.19-2.55)		0.71		N/A		0.00
**MINI^i^-plus^j^**															
	Blended ACT		0		0.10 (0.02-0.30)		N/A		0.09 (0.03-0.21)		N/A		N/A		N/A
	CBT		0		0.02 (0.00-0.17)		N/A		0.08 (0.03-0.19)		N/A		N/A		N/A
**MINI-plus^k^**															
	Blended ACT		1		0.28 (0.14-0.46)		N/A		0.08 (0.02-0.28)		N/A		N/A		N/A
	CBT		1		0.21 (0.10-0.39)		N/A		0.20 (0.05-0.54)		N/A		N/A		N/A

^a^ES: effect size.

^b^GAD-7: Generalized Anxiety Disorder-7.

^c^ACT: acceptance and commitment therapy.

^d^CBT: cognitive behavioral therapy.

^e^MHC-SF: Mental Health Continuum-Short Form.

^f^PHQ-9: Patient Health Questionnaire-9.

^g^SDS: Sheehan Disability Scale.

^h^N/A: not applicable.

^i^MINI: Mini-International Neuropsychiatric Interview.

^j^Probabilities of having an anxiety disorder for participants without anxiety disorder at baseline (n=233).

^k^Probabilities of having an anxiety disorder for participants with anxiety disorder at baseline (n=81).

#### Secondary Outcomes

The T1-T3 by condition interaction was significant for MHC-SF scores (B=.27, *P=*.03, Cohen *d=*0.29): from posttreatment to 12-month follow-up, MHC-SF scores decreased in the CBT group, whereas they increased in the ACT group. For the T0-T1 and T1-T2 intervals, no significant interactions with condition were found, but the significant main effects showed that positive mental health in both groups increased from baseline to posttreatment (B=.29, *P<*.001), and that these improvements were maintained at the month follow-up (B=.00, *P=*.99).

Time-by-condition interactions for PHQ-9 depression and SDS functional impairment were statistically insignificant. Regardless of the condition, depression severity decreased over time, as indicated by the significant main effects for all 3 time intervals (T0-T1 B=−3.01, *P<*.001; T1-T2 B=−.65, *P=*.02; T1-T3 B=−.69, *P=*.04). Functional impairment in work (B=−1.87, *P<*.001), social life (B=−1.78, *P<*.001), and family life (B=−1.93, *P<*.001) significantly decreased from baseline to posttreatment across groups. These decreases were maintained at the month follow-up (work: B=−.18, *P=*.57; social life: B=−.15, *P=*.59; family life: B=−.17, *P=*.51). In both conditions, within-group effect sizes for changes in the MHC-SF, PHQ-9, and SDS during the T0-T1 interval ranged from small to large; those for T1-T2 and T1-T3 were in the very small to small range.

Participants with anxiety disorders at baseline (n=81) had significantly higher baseline GAD-7 scores (mean 10.07, SD 4.09) than participants without an anxiety disorder (mean 1.95, SD 3.85; *F_1_*=16.72, *P<*.001). Among the participants with a baseline anxiety disorder, the odds of meeting the criteria for a disorder at T1 and T3 did not differ significantly between the conditions (T1: B=.38, *P=*.54; T3: B=−1.01, *P=*.35). The odds of participants without a baseline anxiety disorder meeting the criteria for a disorder at T1 and T3 were also not significantly different in the conditions (T1 B=1.28, *P=*.10; T3 B=.05, *P=*.94).

Treatment satisfaction was significantly higher in the ACT group than in the CBT group, and the effect size of the difference was large (B=3.19, *P<*.001, *d=*0.78). No adverse events were reported.

### Improvement and Clinically Significant Change

The proportions of participants with reliable anxiety symptom improvement did not differ significantly between groups (*χ*^2^_1_=0.2, *P=*.66). In the ACT group, 43 of the 101 (42.6%) participants showed reliable improvement at T1. In the CBT group, this was the case for 48 of the 121 (39.7%) participants. In both groups, 2 participants deteriorated. In the ACT group, 22 of the 27 (81.5%) participants with an above-cut-off GAD-7 score at baseline showed clinically significant change, whereas in the CBT group, this was the case for 27 of the 35 (77.1%) participants. These proportions did not differ significantly (*χ*^2^_1_=0.2, *P=*.68).

### PP Analyses

PP analyses included 226 participants (ACT: n=100; CBT: n=126). PP participants did not differ significantly from other participants in terms of baseline characteristics. PP analyses replicated all the findings from the ITT analyses.

## Discussion

This study evaluated the short- and long-term effectiveness of a blended ACT intervention for older adults with mild to moderately severe anxiety symptoms by comparing it with face-to-face CBT. Changes over time in anxiety symptom severity did not differ between the ACT group and CBT group. In both groups, anxiety scores significantly decreased from baseline to posttreatment, and the effect sizes for these decreases were large. At the 12-month follow-up, symptom reduction was maintained in both groups. Furthermore, rates of reliable improvement and clinically significant changes in anxiety symptoms did not differ between the groups. Analyses of secondary outcomes revealed 2 significant differences between the groups. First, improvements in positive mental health were better sustained in the long term in the ACT group. Second, treatment satisfaction was higher for the ACT intervention than for the CBT intervention. No other significant differences in secondary outcomes were found between the groups. Both groups showed significant improvements in depression severity, functional impairment, and positive mental status from baseline to posttreatment, which were mostly sustained or increased at follow-up. Finally, the proportion of participants who met the criteria for a DSM-V anxiety disorder at baseline and no longer did so after treatment did not differ between the ACT group and CBT group.

This was the first large-scale trial to evaluate an ACT intervention for anxiety in later life, and the results therefore strongly contribute to the evidence-based treatment of this highly prevalent and undertreated problem. Overall, the results show that older adults with anxiety symptoms responded similarly to the blended ACT intervention and face-to-face CBT. The insignificant differences between the ACT group and CBT group regarding the majority of outcomes add to null findings from earlier studies comparing ACT and CBT in general adult samples with anxiety symptoms or disorders [54,55]. Therefore, studies thus far have indicated that for anxious adults within a wide age range, ACT and CBT interventions are equally effective. For a more thorough understanding of the (unique) clinical value of blended ACT and face-to-face CBT for anxiety in later life, in subsequent studies we will conduct a cost-effectiveness analysis, examine their working mechanisms (mediator analyses), and determine whether they differentially affect certain subgroups of patients (moderator analyses).

A significant difference between interventions was found for positive mental health: scores from posttreatment to 1-year follow-up decreased in the CBT group and slightly increased in the ACT group. Positive mental health is an important treatment outcome, as studies have shown that after correcting for psychopathology, low levels of positive mental health are associated with more somatic diseases, increased risk of developing a mental disorder, and decreased social and work-related functioning [56]. The interaction effect found in this study is in line with the fact that stimulating people toward value-based and engaged living is an explicit goal of ACT, whereas traditional CBT is primarily focused on alleviating psychopathology [24-26]. However, assuming that ACT directly targets positive mental health, it is unexpected that there was no difference in positive mental health between the groups directly after treatment. Furthermore, the *P* value for the interaction was just below the α level (*P=*.04), and the effect size was small (*d*=0.29). We should, therefore, be careful not to over-interpret this finding. Therefore, the main implication of this finding is that further research into the (long-term) effects of ACT and CBT on positive mental health is warranted.

We found that treatment satisfaction was significantly higher for the blended ACT intervention than for face-to-face CBT. A pilot study on ACT for older adults with anxiety and depressive symptoms found comparable satisfaction ratings [57]. These results suggest that ACT interventions constitute a positive treatment experience for older adults, which could be related to several aspects of the treatment that have been theorized to be especially appealing to this age group [27]. However, these findings need to be interpreted with caution, as treatment satisfaction data were mainly derived from participants who attended all face-to-face sessions. As it is plausible that dropout was associated with lower treatment satisfaction and significantly more participants dropped out in the ACT group, the observed difference might, in part, be the result of selective attrition. We could not rule out this possibility because the data on reasons for dropout were incomplete.

This trial is designed to investigate the relative effectiveness of blended ACT and face-to-face CBT and does therefore not allow conclusions about the absolute effectiveness of the interventions. Still, the significant main effects of time and large within-group effect sizes for anxiety reduction from baseline to posttreatment suggest that both interventions succeeded in treating anxiety symptoms in this sample of older adults. Two earlier trials in anxious older adults found Cohen *d* values of 0.38 and 0.31 for anxiety symptom reduction (measured with the GAD-7) in waitlist conditions [12,13]. The pre-post within-group effect sizes of 0.96 (ACT) and 1.09 (CBT) in this study indicate that the symptom reduction in both conditions greatly surpassed improvements that could have been expected if participants had not received treatment.

The finding that the 2 brief, low-threshold interventions examined in this study were beneficial for a group that currently often goes untreated gives reason to be hopeful. However, to bridge the existing treatment gap, establishing the effectiveness of interventions for anxiety in later life will not suffice; efforts should also be made to increase the uptake of these interventions. In this light, it is promising that this study demonstrated a partial web-based intervention to be equally effective as face-to-face treatment, as scalable internet-based interventions might be crucial in bridging the treatment gap. As the proportion of older adults who successfully use the internet is steadily increasing [58], web-based psychological interventions seem feasible for this age group. However, it is important to note that studies have demonstrated socioeconomic disparities in internet use in older adults—higher education and income levels have been linked to more (successful) internet usage in later life [58]. This was also evident in this trial, in which internet access and basic computer skills were required to participate; more than 85% of the participants had a middle or high level of education. Large-scale implementation of internet-based psychological interventions could therefore increase health inequalities by excluding older adults without internet access or skills from treatment [59]. To improve mental health care in an inclusive manner, studies on the effectiveness and acceptability of psychological interventions for older adults with lower socioeconomic status are needed.

This study has several limitations. First, treatment integrity was assessed suboptimally because it relied on therapists’ self-reports. Second, of the 35,820 people who received the information letter, only 683 registered for study participation; this is a small number considering the high prevalence of anxiety in later life [1-3]. This group is likely to differ from the study population as a whole. For example, all participants were community-dwelling, 98% were of Dutch nationality, and most had middle to high education levels. The generalizability of the findings is also limited because the more severely (psychologically and/or physically) impaired older adults and those over the age of 75 years were excluded from participation. Finally, a considerable number of participants (although comparable with other studies on internet-based and low-threshold or low-intensity interventions in general [60,61]) dropped out before completing treatment, and only one-third of them reported their reason for dropout.

In conclusion, this study is an important advancement in the evidence-based treatment of anxiety in later life. We did not find differences between blended ACT and face-to-face CBT in their effects on anxiety symptom severity and several related clinical outcomes in a large sample of older adults. In both groups, anxiety symptoms improved significantly from baseline to posttreatment, and these improvements had large effect sizes. Regarding the long-term effects on positive mental health, ACT outperformed CBT. Therefore, these findings demonstrate that blended ACT is a valuable treatment alternative to CBT for anxiety in later life, providing patients and therapists with more flexibility in deciding on the preferred intervention with regard to both treatment approach and delivery format. We will follow up this study with examinations of the cost-effectiveness, treatment mediators, and moderators of blended ACT versus CBT. Furthermore, we recommend future research to go beyond the evaluation of psychological interventions for older adults with anxiety symptoms and to focus on increasing treatment uptake in this group.

## Appendix

Multimedia Appendix 1 CONSORT-eHEALTH checklist (V 1.6.1).

## Abbreviations

ACT: acceptance and commitment therapy
CBT: cognitive behavioral therapy
GAD: Generalized Anxiety Disorder
GAD-7: Generalized Anxiety Disorder-7
GP: general practitioner
ITT: intention-to-treat
MHC-SF: Mental Health Continuum-Short Form
MINI-Plus: Mini-International Neuropsychiatric Interview-Plus
PHQ-9: Patient Health Questionnaire-9
PP: per-protocol
RCI: reliable change index
SDS: Sheehan Disability Scale
